# Trichloracetic Acid as a Solvent of Necrosis

**Published:** 1897-12

**Authors:** H. W. Allwine

**Affiliations:** Omaha, Neb.


ARTICLE VI.

### TRICHLORACETIC ACID AS A SOLVENT OF NECROSIS,

BY H. W. ALLWINE, D. D. S., OMAHA, NEB.

Last June Mrs. B. consulted a physician concerning a trouble apparently over, or at the symphysis of the inferior maxillary, and the case was placed in my hands by him. At that time the muscles about the chin were very tender and painful to the least motion, swollen, and of a dark blue color on the outside, as well as about the roots of the teeth. A fistula had formed over the root of the left lateral, from which pus exuded. This tooth was also quite sore to the tooth.

I believed the pulp of this tooth was dead, and proceeded to drill into the lingual surface of the tooth. Before proceeding far I discovered my error. I forced mild antiseptics into the fistula, applied a counter-irritant and dismissed the patient, that I might give the case careful study. In a few days the lady returned. It being a clear day, I noticed a very slight discoloration of the two centrals. These teeth were not in the least sore, and on drilling into them I found the pulps dead. The probe passed through the fistula indicating the unmistakable roughness of necrosis to the touch.

The quickest way out of the difficulty, I thought, was to make an incision and remove the cause. I also suggested to the patient the possibility of dissolving the dental structure with acid, though it might require a long time. She much preferred a fair trial of the latter method, so about once in two weeks I injected a 10 per cent. solution of trichloracetic acid. Twice a week a mild antiseptic was passed into the left central, through the fistula. The apex of the right central was opened nicely, but I could force nothing through the fistula from this tooth. At each visit I packed the canals with cotton saturated with mild antiseptics, and carefully sealed the cavities. I thus treated the case nearly three months. Indications being very favorable, I packed the canals, sealed the cavities, and told the patient to return in two weeks. At this time a very little healthy serum was found in the left central. I treated the case as before, and told the patient to return two weeks later. Then the conditions were such as I wished to have, and I proceeded to put in the fillings. This was done the latter part of September. December 11th I saw the case. The conditions was entirely satisfactory, and I believe a permanent cure was effected.—*Dental Register.*

